# Silica-Based Nanoparticles for Protein Encapsulation and Delivery

**DOI:** 10.3390/nano8110886

**Published:** 2018-11-01

**Authors:** Filippo Begarani, Domenico Cassano, Eleonora Margheritis, Roberto Marotta, Francesco Cardarelli, Valerio Voliani

**Affiliations:** 1 NEST—Scuola Normale Superiore, Pisa 56100, Italy; domenico.cassano@sns.it; 2 Center for Nanotechnology Innovation, Istituto Italiano di Tecnologia, Pisa 56100, Italy; Eleonora.margheritis@iit.it (E.M.); valerio.voliani@iit.it (V.V.); 3 Electron Microscopy Facility, Istituto Italiano di Tecnologia, Genova 00161, Italy; Roberto.marotta@iit.it; 4 NEST—Scuola Normale Superiore, Istituto di Nanoscienze-CNR, Pisa 56100, Italy; francesco.cardarelli@sns.it

**Keywords:** protein-delivery, silica, biodegradation, liposomes, Lysosomal Storage Disorders (LSDs)

## Abstract

Although conceptually obvious, the effective delivery of proteins in therapeutic applications is far from being a routine practice. The major limitation is the conservation of protein physicochemical identity during the transport to the target site. In this regard, nanoparticle-based systems offer new intriguing possibilities, provided that (i) the harsh and denaturating conditions typically used for nanoparticle synthesis are avoided or mitigated; and (ii) nanoparticle biocompatibility and degradation (for protein release) are optimized. Here, we tackle these issues by starting from a nanoparticle architecture already tested for small chemical compounds. In particular, silica-shielded liposomes are produced and loaded with a test protein (i.e., Green Fluorescent Protein) in an aqueous environment. We demonstrate promising results concerning protein encapsulation, protection during intracellular trafficking and final release triggered by nanoparticle degradations in acidic organelles. We believe this proof of principle may open new applications and developments for targeted and efficient protein delivery.

## 1. Introduction

Protein-based therapeutic strategies are currently being actively researched for the treatment of a variety of diseases [1,2,3]. The intrinsic and highly specific functions of proteins, in fact, make their administration the preferred choice for the treatment of diseases characterized, for instance, by the loss of specific enzymatic functions: in this regard, lysosomal storage disorders (LSDs) are probably the best example [4].

Protein-based therapeutic strategies offer, in principle, a unique list of advantages [3,5]. Proteins typically possess a peculiar set of specific roles, which are not easily mimicked by other chemical compounds. This high functional specificity gives protein therapeutics fewer chances to interfere with other off-target biological processes, thus reducing adverse/aspecific responses. In light of these benefits, one may expected reduced times for the clinical approval of protein-based therapeutic compounds as compared to small-molecule drugs [3]. In spite of these advantages, successful protein delivery remains a challenging task. First of all, because, following systemic administration of protein-based compounds, these latter need to overcome a number of biological barriers along the way to their final target. For instance, the loss of protein chemical and physical stability is usually a major challenge. As a consequence, for systemic treatments, proteins are often administered parenterally to avoid the acidic gastro-intestinal tract. In addition, due to the relatively high molecular mass and surface polarity, protein targeted and efficient delivery is inevitably hindered by the presence of biological barriers (e.g., the blood–brain barrier, the endothelium of blood vessels or the cellular plasma membrane [6,7]). Thus far, a number of attempts have been made to tackle these latter limitations, with limited success [5]. Chemical modification of proteins, for instance, although favoring protein circulation half-life, proved to be extremely complex, often ending up in unwanted protein conformational changes (that in turn impair the desired functions [8]). Genetic engineering, such as the creation of Fc-based fusion proteins (made by linking an immunoglobulin Fc domain to the therapeutic protein), greatly contributed to enhancing protein half-life and their therapeutic efficacy. Still, these strategies are often accompanied by an adverse immune response [9]. In this context, a promising role is played by nanoparticle-based systems, thanks to a variety of intrinsic advantageous properties. In fact, nanoparticles are, in general, small enough (<200 nm) to avoid accumulation in spleen and lungs, but large enough (>10 nm) to escape renal clearance [10]. Through surface engineering, nanoparticles can be functionalized with different materials/molecules tailored to different scopes: they can possess biomimetic surfaces [11,12] (stealth systems), pH-responsive moieties [13] or targeting tools, such as antibodies, peptides or small molecules [14]. To be efficiently combined with proteins as cargoes, however, nanoparticle-based strategies must fulfill some additional requirements. In fact, nanoparticle chemical synthesis typically entails using solvents and reagents that can easily affect protein structure/function [15]. Different architectures and materials were tested so far to tackle this crucial limitation. Poly (lactic-co-glycolic acid) (PLGA)-based nanoparticles probably represent the best example in terms of loading efficiency and gentle preparation conditions [5,16,17]. Still, it was shown that PLGA hydrolysis produces an acidic internal environment that can potentially perturb protein activity [18]. On the other hand, virus-derived shells allow for the packaging of several cargos and help to stabilize the platform proving as an efficient approach [19]. Among inorganic nanoparticles, mesoporous silica-based nanoparticles recently emerged as a promising platform in theranostic applications [20,21,22,23,24,25,26]. These nanomaterials present an inert, non-immunogenic, easy-to-modify and biodegradable surface [5,27,28,29]. Regarding their specific application to the protein-delivery field, many attempts were made to combine the benefits of silicon-based materials with the specific needs of a protein-based delivery strategy. Among others, Bégu and co-workers [30] recently designed a system consisting of lipid-based vesicles loaded with both hydrophilic and lipophilic small molecular drugs and then covered with a thin silica shell to increase their stability and allow for surface functionalization. Building on this, we adapted the protocol to the need of encapsulating a protein. Here, in detail, we propose a liposome containing an active protein (in this case, *Aequorea coerulescens* green fluorescent protein, AcGFP) coated with a thin shell of silica that offers a prolonged resistance to acidic, harsh environments. This silica coat will also provide a biodegradable shell allowing the particle (and thus the payload) to reach its target and deliver an active protein or enzyme; the liposome, instead, thanks to its gentle preparation, will allow for the load of the desired protein in its lumen while providing protection during coating. In the following, we shall present the approach for the production and loading of the nanoparticles along with confocal imaging analysis of their trafficking and final fate when administered to living cells.

## 2. Results and Discussion

The synthesis of the proposed nanoparticles began with the formation of liposomes and their loading with AcGFP dissolved in a diethanolamine (DEA) buffer solution (for more details, see Section 4). A thin film of DPPC (1,2-dipalmitoyl-sn-glycero-3-phosphocholine) lipid was first formed following a previously described procedure [31]. The lipid film was then hydrated in DEA buffer (20 mM) solution containing AcGFP (2 µM) through vigorous vortexing and sonication at 40 °C. Once formed and loaded, liposomes were first extruded using a 100 nm pore-size membrane and then centrifuged to remove the supernatant containing the unloaded AcGFP. AcGFP-loaded liposomes were then suspended in fresh DEA buffer solution, and added to an aged tetraethyl orthosilicate (TEOS) solution. The aged TEOS solution was prepared as follows: TEOS was added to deionized water, and the mixture was left under stirring for 24 h at RT. In the aged solution, TEOS is hydrolyzed to orthosilicic acid and, when its concentration exceeds the saturation limit, its polymerization occurs [32]. The process yields, in sequence, low to high-molecular polymers and, due to their condensation, silica particles of 1–2 nm in diameter. AcGFP-loaded liposomes were added to the aged solution before silica nuclei increase in size following a LaMer growth pattern, resulting in their absorption on the surface of liposomes by ionic interactions. Then, the remaining orthosilicic acid continues to form a complete shell until its saturation limit is reached or the reaction interrupted. Silica-protected AcGFP-loaded liposomes were finally purified by the mother solution by centrifugation. It is worth mentioning that the entire process is performed in physiological pH water solutions (please note that different buffers other than DEA can be employed; see Section 4), in order to preserve the activity of the loaded proteins.

**Figure 1:**
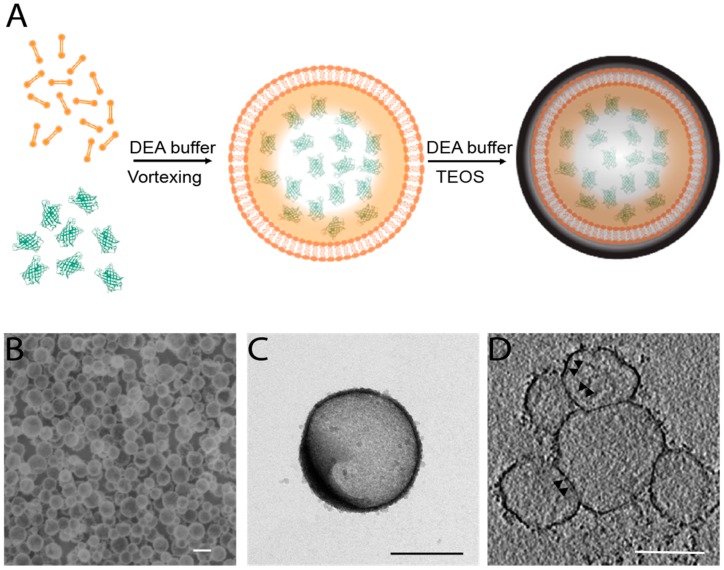
Schematic representation of nanoparticle synthesis (**A**). SEM characterization of the obtained nanoparticles, scale bar 200 nm (**B**). TEM image of a single *Aequorea coerulescens* green fluorescent protein (AcGFP)-loaded nanoparticle, scale bar 200 nm (**C**). Averaged tomographic slice of several AcGFP-loaded nanoparticles. Black arrows point to the lipid layer present underneath the silica layer (darker contour) that delimit the nanoparticles, scale bar 100 nm (**D**).

**Figure 2:**
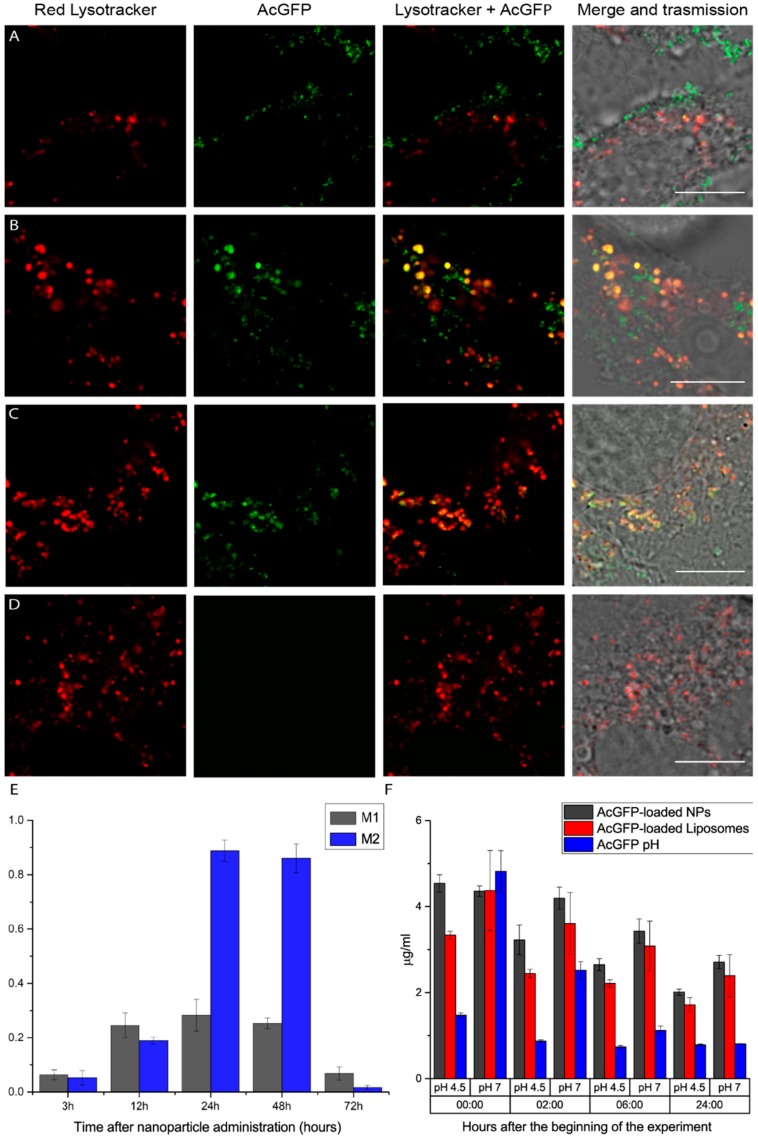
Confocal images for colocalization analysis (**A**–**D**). Columns on the left show the red Lysotracker channel, while middle columns show the AcGFP-loaded nanoparticle channel and columns on the right are simply the merge of Lysotracker and protein channels. Rows represents the situation at different time points: 3 h (**A**), 12 h (**B**), 24 h (**C**) and 72 h (**D**) after particle administration. Please note how particles all colocalize with lysosomes at 24 h and at 72 h; no sign from AcGFP was detected. Scale bar: 10 µm. (**E**) Manders’ colocalization coefficients; referring to M1 as the colocalization of the AcGFP channel with the Lysotracker channel and vice versa for M2. Colocalization rallies to a maximum at 12 and 24 h after particle administration and steeply decreases at 72 h. (**F**) Amount of active protein in both citric acid (pH 4.5) and PBS (pH 7) solutions at different time points (namely, time 0, and 2, 6 and 24 h since the beginning of the experiment). Black bars: AcGFP-loaded nanoparticles. Red bars: AcGFP-loaded (1,2-dipalmitoyl-sn-glycero-3-phosphocholine) (DPPC) liposomes. Blue bars: free AcGFP in solution. The initial quantity of protein is equal for each single condition. Please note how black bars (i.e., protein-loaded nanoparticles) maintain higher levels especially for the later points.

Figure 1A briefly summarizes the concept. SEM, TEM and especially CryoEM characterizations reported in Figure 1B–D show the obtained nanoparticles with an external thin layer (~2–3 nm) of silica and average diameters of 137 ± 40 nm (reported as mean ± standard deviation, see Figure S1). CryoEM also allowed us to recognize the lipid bilayer just underneath the silica coat (Figure 2D; for more details see Figure S2 and Video S1), further supporting the final architecture of the nanosystem.

Dynamic light scattering (DLS) measurements returned an average hydrodynamic diameter (HD) size of about 390 ± 46 nm (average ± SD) with a quite low polydispersity index (PI = 0.2) and a zeta-potential of −17.2 ± 3.4 mV. It is worthwhile to remember that HD values are typically bigger with respect to the diameter estimate extracted from TEM or SEM because HD comprises both the actual diameter of the nanoparticles and the solvation layer. Please note that the surface of silica-coated liposomes can be easily modified with the addition of (3-amminopropyl)triethoxysilane (APTES) to change the surface electrostatic charge of nanoparticles and add anchor points for the attachment of moieties of interest. APTES functionalization was achieved by adding 5 µL of APTES in 1.5 mL of water containing AcGFP-loaded liposomes; the solution was then left under gentle stirring for 15 min at 37 °C. This was confirmed by a positive zeta-potential value being measured (15.1 ± 4.1 mV).

To assess encapsulation efficiency, we derived a calibration curve based on the fluorescence intensity recorded at different protein concentrations (exploiting AcGFP intrinsic fluorescence) and compared the calibration curve with the results obtained in the nanoparticle samples (see Section 4). We estimated an encapsulation efficiency of about 10%. Although not high, the experimental conditions used during the whole encapsulation process are compatible with the recovery of non-encapsulated proteins (and their possible re-use, thus avoiding the waste of material).

Cell studies were conducted in HeLa cells (Figure 2). AcGFP-loaded nanoparticles were suspended in the cell-culture medium without serum and cells were incubated for 3 h. After incubation, cells were washed with PBS three times and the complete cell-culture medium with Lysotracker was added following the standard protocol. Nanoparticle colocalization with Lysotracker was assessed using confocal microscopy at different time points (namely 3, 12, 24, 48 and 72 h) following their administration. The data showed a peak of colocalization inside lysosomes 24 h after administration, while at 72 h we were not able to recover any fluorescent signal originating from AcGFP. Manders’ coefficients (Figure 2E) also quantitatively show that colocalization starts to increase at 12 h, reaching a peak at 24 h and 48 h, and then decreases suddenly at 72 h. We interpreted this result as the consequence of nanoparticle degradation inside lysosomes, followed by the further digestion of AcGFP protein. Of particular note, the tested nanosystems do not elicit cytotoxicity in this set-up (Figure S3).

To study nanoparticle and protein stability in acidic conditions, which are typical of the lysosomal environment (with pH values included in the 4.8–4.5 range) [33], we designed an experiment based on monitoring AcGFP fluorescence emission (and, as a consequence, protein stability) in different buffer conditions at 37 °C (Figure 2F). We added the nanoparticles into two different solutions: citric acid (pH 4.5) and PBS (pH 7). Liposomes containing the same amount of protein as the tested nanoparticles and isolated AcGFP were used as controls. Fluorescence intensities from the six different conditions were collected for 24 h at different time points (0, 2, 6 and 24 h after the beginning of the experiment). The experiment showed how the proposed nanoparticles help to protect their payload for the longest time, while protein alone, especially at acidic pH, gets denatured in a short time. Compared to liposomes, it is easy to note that silica-based nanoparticles maintain a higher intrinsic fluorescence intensity (thus, a higher amount of active protein) for all time points, for both acidic and neutral conditions. We can see how the amount of fluorescence signal from the nanoparticles at the zero-time point is equal for both acidic and neutral conditions (gray columns on the left, Figure 2F) while for liposomes and proteins alone, the acidic conditions show lower signal values compared to the neutral cases (red and blue columns on the left, Figure 2F). This supports the idea that the silica shell effectively provides a shield against acidic solutions, protecting the stability and functionality of the protein loaded. This interpretation is also supported by the studies conducted in cells using AcGFP-loaded liposomes alone (Figures S4 and S5). In this case, liposomes lasted inside cells only for 6–8 h and they never reached such a high internalization efficiency (despite a zeta-potential of 2 mV), thus further proving the validity of our initial assumptions about the proposed architecture.

It is worth noticing that, during the entire duration of cell experiments, cell cultures were not washed following the first time point studied (that is, 3 h after administration; see Section 4) and the presence of nanoparticles was not detected outside cells (as also confirmed by Manders’ coefficients). This evidence, taken together with the tests conducted at different pH conditions that showed that particles in both physiological and acidic environments maintain their fluorescence for more than 24 h, supports the hypothesis of particle degradation inside lysosomes. Furthermore, these findings are in agreement with the biodegradation of similar silica-based nanoparticles [25].

## 3. Conclusions

Overall, the combination of nanoparticle architecture and a mild synthesis environment can represent a potential effective approach which is not limited to small organic molecules and open new perspectives for protein encapsulation and delivery. Functionalization with effective targeting molecules is a further step to be addressed, along with the encapsulation of different proteins, tailored to specific biomedical applications. With regard to targeting, it is worth noting that the present nanosystem has already proved its ability to efficiently target the lysosomes, with no need for additional modifications. As we speculated, the degradation of the silica shell inside lysosomes (with the subsequent release of intact protein cargoes) may represent an interesting opportunity for the treatment of LSDs: the delivery of the active galactoceribrosidase (GALC) enzyme to lysosomes as an enzyme replacement therapy (ERT) represents, for instance, the preferred choice of treatment for the rare Krabbe disease [34]. In general, the architecture and results herein reported represent only the beginning of an itinerary required for their validation as a potent tool for protein delivery and therapeutics.

## 4. Materials and Methods

### 4.1. Synthesis of GFP-Loaded Nanoparticles

#### 4.1.1. GFP-Loaded Liposome Preparation

DPPC, 1,2-dipalmitoyl-sn-glycero-3-phosphocholine (10 mg/mL in chloroform) lipid was purchased from Avanti Polar Lipids (Alabaster, AL, USA). One hundred microliters of chloroform solution containing 1 mg of DPPC was placed in a centrifugal evaporator under vacuum for 2 h to obtain a lipid thin film. Four hundred microliters of 20 µM DEA buffer solution (suitable for AcGFP stability) containing AcGFP at a concentration of 250 µM was poured onto the lipid film, which was hydrated through vigorous vortexing and sonication at 40°, thus creating a gentle condition for protein stability conservation. The final DPPC concentration was 2.5 mg/mL. At this point, liposomes were extruded using an extruder (Avanti Polar Lipids, Alabaster, AL, USA) equipped with membranes of polycarbonate. Three different pore-size membranes were used in sequence: 400, 200 and 100 nm as average size diameters. The extrusion was performed at 40 °C to keep the lipid at a temperature higher than its transition phase temperature but in a viable range for protein function preservation. Once extruded, liposomes were centrifuged at 38,000 rcf for 3 h and, after this step, the supernatant containing the unloaded protein was removed and concentrated to be used again for other synthesis. The precipitated liposomes were instead suspended in 400 µL of DEA buffer solution (20 µM) to obtain a lipid final concentration of 2.5 mg/mL. Liposomes created following this latter procedure were also employed to perform control experiments.

#### 4.1.2. Silica Coating Formation

Twelve microliters of TEOS (thetraethyl orthosilicate, Sigma-Aldrich, Saint Louis, MO, USA) were poured into 1 mL of deionized water, and the solution was kept in an agitator (gentle agitation) for 24 h at 40 °C. The day afterwards, 20 µL of the liposome suspension obtained as described above was added to the 1 mL TEOS solution and the mixture was kept in gentle agitation at 21 °C for 1 h. At this point, 2 mL of DEA buffer (20 mM) solution was added to the mixture along with 24 µL of fresh TEOS and everything was left in agitation at 21 °C for 24 h. It is important to notice that the same results can be obtained using PBS (1×) instead of DEA; if PBS is to be used, 36 h (instead of 24) are required to complete the entire process. The day afterwards, the final product was washed using repeated cycles of centrifuge (10,500 rcf for 5 min) and deionized water rinse. The precipitate consisted of the silica-coated liposomes.

### 4.2. APTES Functionalization

Once nanoparticle synthesis was completed, 0.5 µL of (3-amminopropyl)triethoxysilane (APTES, Sigma-Aldrich, Saint Louis, MO, USA) was added to 1.5 mL of deionized water containing nanoparticles (the amount corresponded to the outcome of one synthesis described above). The newly made solution was left in gentle agitation for 15 min at 37 °C. After that, nanoparticles were washed through repeated cycles of centrifuge (10,500 rcf for 5 min) and deionized water.

### 4.3. Dynamic Light Scattering (DLS) Measurements

DLS and ζ-potential measurements were carried out using a Malvern Instruments (Malvern, UK) Zetasizer Nano Z. Particles were measured while suspended in 1 mL of deionized water. The values of the hydrodynamic radius and Z-potential were derived from 5 subsequent measurements.

### 4.4. Cell Culture and Treatments

HeLa cells (CCL-2 ATCC) were cultured in Dulbecco’s modified Eagle medium (DMEM) without phenol red (Gibco, Thermo Fisher Scientific, Waltham, MA, USA), supplemented with 10% fetal bovine serum (FBS, Gibco, Thermo Fisher Scientific, Waltham, MA, USA), 100 U/mL of penicillin, and 100 μg/mL of streptomycin in a humidified incubator at 37 °C and 5% CO_2_. Cells were seeded on 22-mm glass bottom dishes (WillCo Wells, Amsterdam, The Netherlands) and allowed to adhere overnight in a 37 °C and 5% CO_2_ cell culture incubator.

For nanoparticle internalization analysis, cells previously seeded on 22-mm glass bottom dishes were washed three times with PBS (1×) and then were incubated for 3 h in growing medium without serum, but containing 10 µg/mL of nanoparticles (or liposomes, for control experiments). After 3 h, cells were again washed three times with PBS and then incubated with culture medium with serum. At 3, 6, 12, 16, 24, 48 and 72 h after particle administration cells received 60 µL of 1 µM of LysoTracker Red DND-99 (Invitrogen, Carlsbad, CA, USA) as the protocol suggests and were analyzed under a confocal microscope. After the initial 3 h of incubation with particles, cells were not washed anymore to avoid the removal of nanoparticles not internalized by the cells.

### 4.5. Live-Cell Imaging and Co-Localization Experiments

Co-localization imaging experiments were carried out on a FV1000 confocal microscope (Olympus, Tokyo, Japan) equipped with a 60× NA 1.2 water immersion objective. AcGFP was excited using a 488 nm laser line, and its emission was collected in the 490–540 nm range, while Lysotracker red was excited using 543 nm laser line and its signal was collected in the 550–650 nm range.

Colocalization analysis between AcGFP and Lysotracker channels was carried out using the ‘Coloc 2’ plugin contained in the latest version of Fiji (ImageJ, NIH, Bethesda, MD, USA). Regarding the results, only Manders’ colocalization coefficients were considered, and in particular, the threshold coefficients, tM1 and tM2. Referring to tM1 as the colocalization of GFP channel with Lysotracker channel and vice versa for tM2.

### 4.6. Cytotoxicity Assay

Cytotoxicity assay was performed on HeLa cells using the Cell Counting Kit-8 (CCK-8, Sigma-Aldrich, Saint Louis, MO, USA). Four different conditions were analyzed and each experiment was conducted in triplicate. As a positive control, cell viability was tested on simple HeLa cell culture; as negative control, cells were also cultured with medium containing 10% DMSO (Dimethyl sulfoxide). Along with controls, cells received three different amounts of AcGFP-loaded nanoparticles to test their cytotoxicity (that is, 1, 10 and 20 µg of nanoparticles per well). The experiment was conducted following exactly the provider’s instructions for each time point tested (3, 24 and 72 h since treatment administration). Cells that received the nanoparticles were cultured and treated as described above, while cells with DMSO were only washed three times with PBS and, after that, 10% DMSO growing medium was added to the cultures. For each well (and thus each case), 7500 cells were seeded.

### 4.7. Calibration Curve for Encapsulation Efficiency Analysis

To assess the protein-encapsulation efficiency, the fluorescence intensities of solutions at different AcGFP concentrations were acquired. AcGFP were diluted in DEA buffer (20 mM) to obtain four different concentrations: 2.5, 25, 250, 2500 nM. This calibration study was conducted using a Varian Cary Eclipse Fluorescence Spectrophotometer (Agilent Technologies, Santa Clara, CA, USA). A linear calibration fit was obtained that allowed for the direct measurement and quantification of the loaded AcGFP inside nanoparticles. In brief, knowing the starting protein concentration (and thus the quantity) used and the concentration left in the supernatant at the end of the synthesis process allowed us to find an indirect esteem of the protein quantity inside the particles that perfectly matched the quantity measured directly. Please note that measuring intensity fluorescence allowed us to study and quantify only active proteins.

### 4.8. Protein Degradation Study Experiment

To assess protein degradation, the same number of nanoparticles (100 µg/mL) was added to 2 different buffer solutions: PBS (pH 7) and citric acid (pH 4.5). Samples were kept under gentle stirring for 24 h. At specific time points (0, 2, 6 and 24 h), solutions were placed under the fluorimeter (Varian Cary Eclipse Fluorescence Spectrophotometer, Agilent Technologies, Santa Clara, CA, USA) to measure the AcGFP fluorescent signal. Results were plotted to show and compare the intensity signals proportional to the amount of active protein present. Control experiment using the same amount of AcGFP were also conducted with AcGFP-loaded DPPC liposomes (100 µg/mL) and AcGFP free protein (in this case, the amount of protein used was calculated following the results of encapsulation efficiency of nanoparticles in order to have the same amount of protein for all the six conditions tested).

### 4.9. TEM and SEM Imaging

TEM images were obtained with a ZEISS Libra 120 TEM (Carl Zeiss, Oberkochen, Germany) operating at an accelerating voltage of 120 kV. Samples were prepared by inserting a carbon-coated 300-mesh copper TEM grid into an aqueous suspension containing the particles and analyzed with TEM microscope 3 h later following solvent evaporation.

SEM images were obtained with a ZEISS Merlin SEM (Carl Zeiss, Oberkochen, Germany) operating at an acceleration voltage of 5 kV. Samples were prepared on a Silicon wafer substrate and a drop of an aqueous suspension containing the particles was left to dry on it for 24 h to let solvent evaporate.

### 4.10. Diameter Size Distribution

Measurements of the diameter of nanoparticles were conducted using Fiji software (ImageJ, NIH, Bethesda, MD, USA) (https://imagej.net/Fiji) starting from SEM images acquired as described above.

### 4.11. CryoEM and Cryo Electron Tomography Imaging

Samples were vitrified by applying a 3 μL aliquot to previously glow-discharged 200-mesh holey and Quantifoil carbon grids (Ted Pella, Redding, CA, USA). Grids were blotted and then plunged into liquid ethane using an FEI Vitrobot Mark IV (FEI Company, Eindhoven, The Netherlands). The samples were imaged in bright field transmission electron microscopy (TEM) using a Tecnai G2 F20 microscope (FEI Company, Eindhoven, The Netherlands) equipped with a Schottky field emission electron source and a US1000 2k × 2k Gatan CCD camera operating at an acceleration voltage of 200 kV. The cryo-tomographic tilt series were collected tilting the vitrified sample over ±60°, with a tilt step of 2°. The computation of the tomogram was carried out with the IMOD28 software package. The cryo-EM and cryo electron tomography CET imaging was performed under low-dose conditions (with a total dose of 1.5 electrons/Å2 for single projection images and 60–80 electrons/Å2 for a whole tilt series).

### 4.12. AcGFP Protein Expression and Purification

Protein expression and purification were performed as described by Storti et al. [35]. Briefly, the sequence encoding for *Aequorea coerulescens* green fluorescent protein was cloned from the pAcGFP1—N1 vector (Clontech, Mountain View, CA, USA) into the pET28c expression vector, between NdeI and NotI restriction sites, and transformed in *E. coli* BL21 (DE3) strain (Invitrogen, Carlsbad, CA, USA).

Cells were grown at 37 °C to an absorbance at 600 nm of 0.6, and protein expression was induced with 250 μM Isopropyl β-d-1-thiogalactopyranoside (IPTG—Sigma) at 28 °C for 16 h. Cells were harvested by centrifugation (4500× *g*, 20 min, 4 °C) and frozen at −20 °C. Cells were then resuspended in ice-cold lysis buffer A: 50 mM tris-HCl pH 8.0, 150 mM NaCl, EDTA-free protease inhibitor cocktail (Roche, Basel, Switzerland) and lysed by sonication on ice, followed by 1 h treatment with 0.1% Triton-X100 at 4 °C. After the removal of the debris by centrifugation (12,000× *g*, 1 h, 4 °C), the supernatant was mixed with 5 mL of NiNTA Agarose beads (QIAGEN) and incubated on a rotor for 4 h at 4 °C. The His-tagged protein was then eluted in buffer A + 500 mM Imidazole and dialyzed against buffer B: 20 mM diethanolamine (DEA) pH 8.5. Protein purity was finally evaluated by SDS-PAGE, and the concentration was determined by UV absorption measurements.

## 5. Statistical Analysis

Quantities studied for this research work were all normally distributed. Thus, their values are reported throughout the text as Mean ± SD, and compared among different experiments using the Student’s *t* test.

## Supplementary Materials

The following are available online at http://www.mdpi.com/2079-4991/8/11/886/s1, Figure S1: Diameter size distribution of nanoparticles, Figure S2: Cryo-EM projection image of several AcGFP-loaded nanoparticles, Figure S3: Cytotoxicity assay performed in HeLa cells, Figure S4: Confocal images for colocalization analysis of control experiment conducted with AcGFP-loaded DPPC liposomes, Figure S5: Manders’ colocalization coefficients, Video S1: 3D reconstruction of cryo tomography of AcGFP-loaded nanoparticles.
