# Supplementary material for: Silica-Based Nanoparticles for Protein Encapsulation and Delivery
Source: Nanomaterials (Basel). 2018 Nov 1;8(11):886. doi: 10.3390/nano8110886 (PMC6266174; doi:10.3390/nano8110886)
Supplement: Supplementary file 1 [file nanomaterials-08-00886-s001.zip › nanomaterials-380754-SI.pdf]

Silica-Based Nanoparticles for Protein Encapsulation and Delivery

F. Begarani [1,2,*], D. Cassano [1,2], E. Margheritis [2], R. Marotta [3], F. Cardarelli [4] and V. Voliani [2]

[1] NEST—Scuola Normale Superiore, Pisa, Italy; domenico.cassano@sns.it
[2] Center for Nanotechnology Innovation, Istituto Italiano di Tecnologia, Pisa, Italy; Eleonora.margheritis@iit.it (E.M.); valerio.voliani@iit.it (V.V.)
[3] Electron Microscopy Facility, Istituto Italiano di Tecnologia, Genova, Italy; Roberto.marotta@iit.it
[4] NEST—Scuola Normale Superiore, Istituto di Nanoscienze-CNR, Pisa, Italy; francesco.cardarelli@sns.it
* Correspondence: filippo.begarani@sns.it; Tel.: +39-050-509-563

Supplementary Information

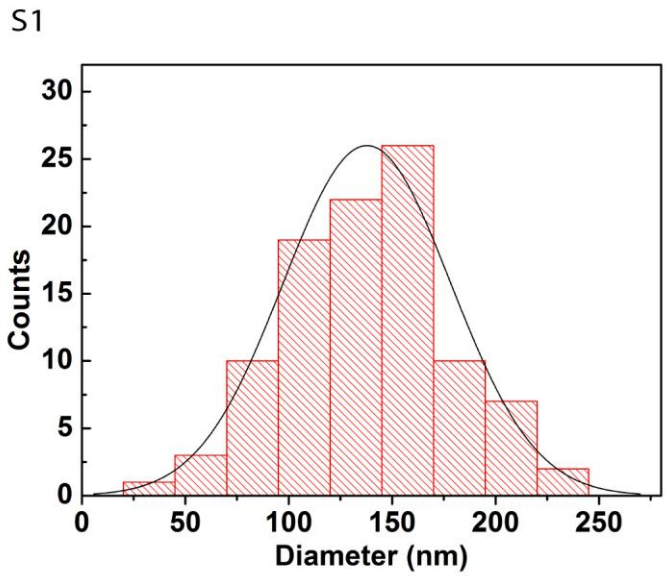

Figure S1. Diameter size distribution of nanoparticles. Diameter size distribution of nanoparticles obtained from SEM images that returned a value of 137 ±40 nm (presented as mean ± standard deviation).

S2

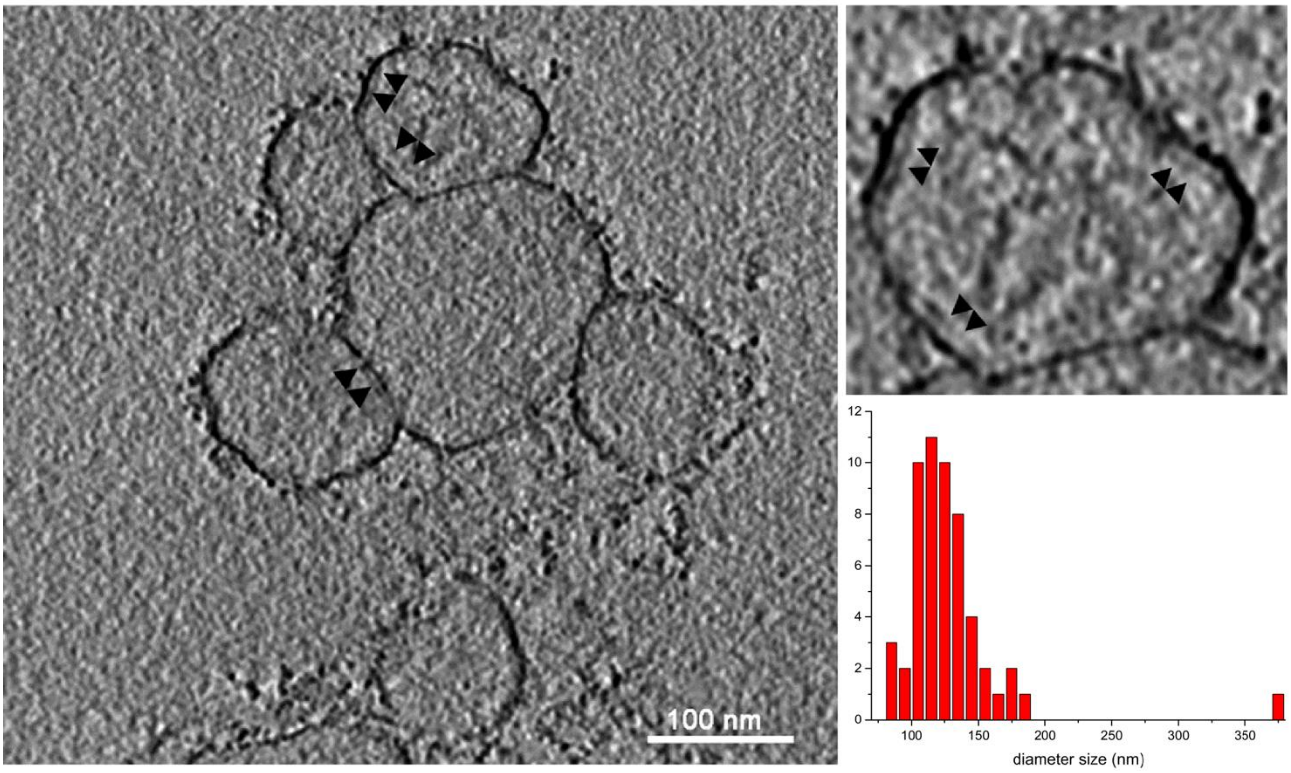

Figure S2. Cryo-EM projection image of several AcGFP-loaded nanoparticles. Cryo-EM projection image of several AcGFP-loaded nanoparticles (a). Averaged cryo-tomographic slice of the same AcGFP-loaded nanoparticles imaged in (a). Black arrows point to the lipid layer present underneath the silica layer (darker contour) that delimit the nanoparticles. On the upper right, a magnification of the picture on the left is reported. On the lower right, a size distribution of particle diameters is shown, resulting in an average value of 128±39 nm (mean ± SD) over a total of 70 particles analyzed. It is worth to mention that sample preparation partially damaged nanoparticles, explaining why they do not show the perfect round shape reported in SEM and TEM images.

S3

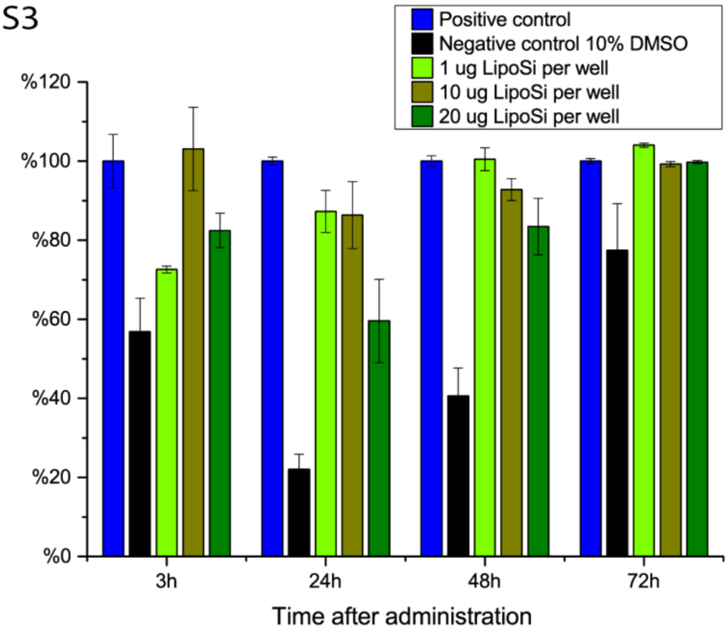

Figure S3. Cytotoxicity assay performed in HeLa cells. Four different conditions at different time points (namely, 3, 24, 48 and 72 hours after particle administration) were tested. Blue bars: HeLa cell cultures following standard procedures (positive control). Black bars: Hela cells that received 10% DMSO growing medium (negative control). Light green bars: HeLa cells that received 1µg of nanoparticle. Brown-green bars: HeLa cells that received 10 µg of nanoparticles. Dark green bars: HeLa cells that received 20 µg of nanoparticles. It is worth to highlight that nanoparticles did not show a high degree of cytotoxicity, this is testified by the fact that at 72 hours the amounts of living cells in the samples that received nanoparticles were in line with the positive control for all three particle concentrations.

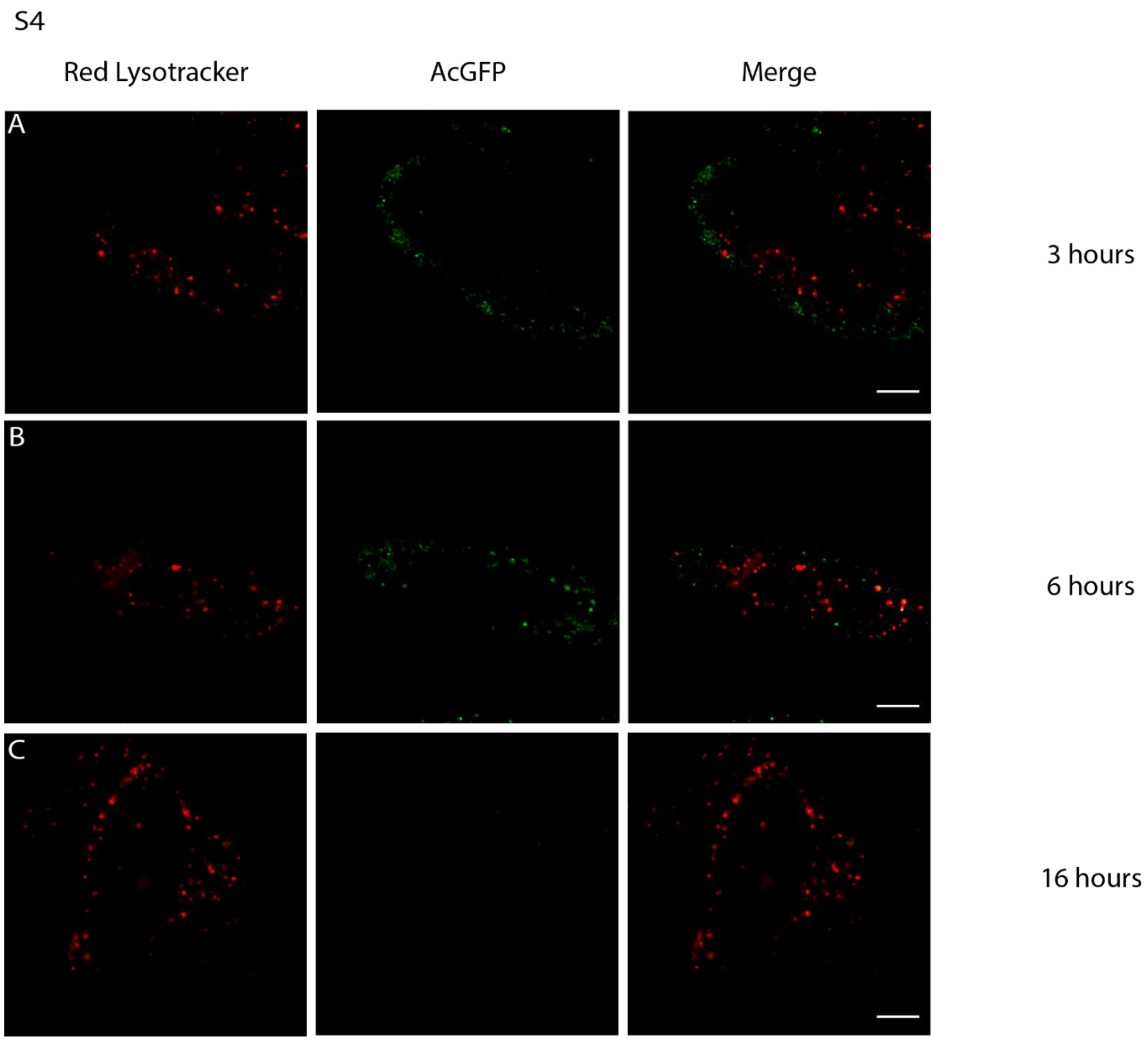

Figure S4. Confocal images for colocalization analysis of control experiment conducted with AcGFP-loaded DPPC liposomes. Columns on the left report Red Lysotracker channel, while middle columns show AcGFP-loaded liposome channel and columns on the right are simply the merge of Lysotracker and protein channels. Rows represents the situation at different time points: 3 hours (A), 6 hours (B) and 16 hours (C) after liposome administration. Scale bar 10 µm.

S5

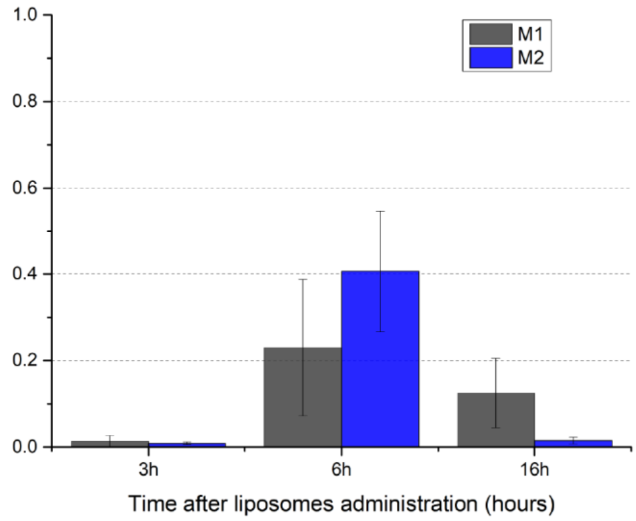

Figure S5. Manders' colocalization coefficients. Referring to M1 as the colocalization of AcGFP channel with Lysotracker channel and vice versa for M2. Colocalization increases at 6 hours after particle administration and steeply decreases at 16 hours. Although, colocalization was detected, Manders' coefficients did not reach the high value of silica nanoparticle experiments (Figure 3A), thus suggesting how liposomes alone also possess a lower internalization efficiency.

Supplementary Movie

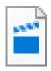

Movie_S1.mp4

3D reconstruction of Cryo-tomography acquired on the same AcGFP-loaded nanoparticles imaged in S2.
